# Retraction: Integrative bioinformatics approaches to map key biological markers and therapeutic drugs in Extramammary Paget’s disease of the scrotum

**DOI:** 10.1371/journal.pone.0273532

**Published:** 2022-08-31

**Authors:** The PLOS ONE Editors

The *PLOS ONE* Editors retract this article [1, 2] because it was identified as one of a series of submissions for which we have concerns about authorship, competing interests, and peer review. We regret that the issues were not addressed prior to the article’s publication.

All authors did not agree with the retraction.

## References

[1] Noor F, Saleem MH, Chen J-T, Javed MR, Al-Megrin WA, Aslam S (2021) Integrative bioinformatics approaches to map key biological markers and therapeutic drugs in Extramammary Paget’s disease of the scrotum. PLoS ONE 16(7): e0254678. 10.1371/journal.pone.0254678 34292991 PMC8297842

[2] Noor F, Saleem MH, Chen J-T, Javed MR, Al-Megrin WA, Aslam S (2021) Correction: Integrative bioinformatics approaches to map key biological markers and therapeutic drugs in Extramammary Paget’s disease of the scrotum. PLoS ONE 16(10): e0259408. 10.1371/journal.pone.0259408 34705874 PMC8550377

